# Development and In Vitro Evaluation of Biocompatible PLA-Based Trilayer Nanofibrous Membranes for the Delivery of Nanoceria: A Novel Approach for Diabetic Wound Healing

**DOI:** 10.3390/polym13213630

**Published:** 2021-10-21

**Authors:** Mohamed Ahmed Mohamady Hussein, Sena Su, Songul Ulag, Agata Woźniak, Mariusz Grinholc, Gökce Erdemir, Serap Erdem Kuruca, Oguzhan Gunduz, Mamoun Muhammed, Ibrahim M. El-Sherbiny, Mosaad Megahed

**Affiliations:** 1 Clinic of Dermatology, University Hospital of RWTH Aachen, 52074 Aachen, Germany; mmegahed@ukaachen.de; 2 Department of Pharmacology, Medical Research Division, National Research Center, Dokki, Cairo 12622, Egypt; 3 Center for Nanotechnology & Biomaterials Application and Research (NBUAM), Marmara University, Istanbul 34722, Turkey; senasu01@gmail.com (S.S.); ulagitu1773@gmail.com (S.U.); oguzhan@marmara.edu.tr (O.G.); 4 Laboratory of Molecular Diagnostics, Department of Biotechnology, Intercollegiate Faculty of Biotechnology, University of Gdansk, 80-307 Gdansk, Poland; agata.wozniak@phdstud.ug.edu.pl (A.W.); mariusz.grinholc@biotech.ug.edu.pl (M.G.); 5 Department of Molecular Medicine, Aziz Sancar Institute of Experimental Medicine, Istanbul University, Istanbul 34390, Turkey; gokcerdemir@gmail.com; 6 Molecular Cancer Research Center (ISUMKAM), Istinye University, Istanbul 34010, Turkey; 7 Department of Physiology, Faculty of Medicine, Istanbul University, Istanbul 34390, Turkey; sererdem@yahoo.com; 8 Department of Metallurgical and Materials Engineering, Faculty of Technology, Marmara University, Istanbul 34722, Turkey; 9 KTH Royal Institute of Technology, SE-100 44 Stockholm, Sweden; mamoun@kth.se; 10 Nanomedicine Laboratory, Center for Materials Science (CMS), Zewail City of Science and Technology, Giza 12578, Egypt

**Keywords:** nanoceria, PVA, PLA-based trilayer nanofibrous membranes, fibroblasts, diabetic wound healing

## Abstract

The attempts to explore and optimize the efficiency of diabetic wound healing’s promotors are still in progress. Incorporation of cerium oxide nanoparticles (nCeO_2_) in appropriate nanofibers (NFs) can prolong and maximize their promoting effect for the healing of diabetic wounds, through their sustained releases, as well as the nanofibers role in mimicking of the extra cellular matrix (ECM). The as-prepared nCeO_2_ were analyzed by using UV–Vis spectroscopy, XRD, SEM–EDX, TEM and FTIR, where TEM and SEM images of both aqueous suspension and powder showed spherical/ovoid-shaped particles. Biodegradable trilayer NFs with cytobiocompatibility were developed to sandwich nCeO_2_ in PVA NFs as a middle layer where PLA NFs were electrospun as outer bilayer. The nCeO_2_-loaded trilayer NFs were characterized by SEM, XRD, FTIR and DSC. A two-stage release behavior was observed when the nanoceria was released from the trilayer-based nanofibers; an initial burst release took place, and then it was followed by a sustained release pattern. The mouse embryo fibroblasts, i.e., 3T3 cells, were seeded over the nCeO_2_-loaded NFs mats to investigate their cyto-biocompatibility. The presence and sustained release of nCeO_2_ efficiently enhance the adhesion, growth and proliferation of the fibroblasts’ populations. Moreover, the incorporation of nCeO_2_ with a higher amount into the designed trilayer NFs demonstrated a significant improvement in morphological, mechanical, thermal and cyto-biocompatibility properties than lower doses. Overall, the obtained results suggest that designated trilayer nanofibrous membranes would offer a specific approach for the treatment of diabetic wounds through an effective controlled release of nCeO_2_.

## 1. Introduction

It is documented that about 422 million people (8.5% of the population of the world), are suffering from diabetes mellitus. It is estimated that 15% of patients with diabetes are likely to be affected with diabetic wound; meanwhile, patients with a diabetic wound are mostly facing the risk of amputations of their lower extremity [1,2]. As a result of high incidence of diabetes, with no efficient treatments, amputation of a part or a whole lower limb occurs every 30 s somewhere in the world [3]. Patients with foot diabetic ulcers complain of severe pains and a poor quality of life [4]. Overexpression of reactive oxygen species (ROS) in the wound environment and, thus, the impairment of wound healing are the major health concerns in diabetes patients [5,6]. The current trends are focusing on developing hybrid nanoparticulate systems, such as ROS-scavengers for the alleviation of oxidative stress wounds [7,8].

Cerium oxide nanoparticles (nanoceria, nCeO_2_) have been reported as a promising nanomaterial to induce wound healing [9]. Nanoceria was reported as being a non-toxic material, and it was stated that nCeO_2_ showed a non-toxic effect on the fibroblast populations [10]. Many studies confirmed that nanoceria displayed non-toxic action when it was applied on the biological tissues [11]. Thus, nanoceria was introduced widely in biological systems as a neuroprotective, cardioprotective, radiation-protective and tissue regenerative agent [12,13,14,15]. Topical application of nCeO_2_ in full-thickness dermal wounds in mice was found to enhance the proliferation and migration of fibroblasts, keratinocytes and vascular endothelial cells (VECs) [16]. Moreover, nanoceria has the ability to penetrate into cells, as well as good antioxidant properties because of its capability to scavenge reactive oxygen species (ROS). Therefore, nanoceria has received a great amount of attention in various biological applications, such as optical biosensors for radicals [17], cancer treatment [18], neuro-protection during spinal cord repairing [19], angiogenesis enhancement [20] and treatment of chronic inflammation through inhibiting inflammatory mediator production [21].

The fraction of Ce^3+^ on the surface of nanoceria is compensated by abundant oxygen vacancies, and enables it to mimic the key action of antioxidant enzymes, such as superoxide dismutase (SOD) to scavenge ROS. Moreover, the spontaneous switch of +3/+4 valence allows nanoceria to recycle its antioxidant action and act as an antioxidant never-ending machine, as in redox enzymes [22].

In a murine model of cardiomyopathy, nanoceria could suppress the progression of cardiac dysfunction, and this was attributed to the auto-regenerative antioxidant properties of nanoceria [13]. In an in vivo study that used nanoceria (250 μg/mL) incorporated into gelatin hydrogel, a wound was healed in a shorter amount of time than a wounded rat treated with neat gelatin. It has been found that leukocytes’ infiltration and collagen deposition have occurred in higher ratios in wounds treated with gelatin–cerium oxide nanocomposite [23]. The same results have been obtained by using ceria, but at a higher concentration of 2% [24].

Nanofibers (NFs) are produced via electrospinning technique as randomly oriented structures. NFs are applied for tissue regeneration, due to their physical, mechanical and morphological characteristics. The porosity structure of NFs is necessary to facilitate the mobility of nutrients and oxygen for the cells’ needs [25]. The structural similarity of the electrospun NFs to ECM gives them a high priority to be used as a substrate for wound dressing. Polyvinyl alcohol (PVA), a synthetic, non-toxic and biocompatible polymer, used widely in tissue engineering and wound dressing [26,27]. Poly (lactic acid) (PLA), is a biocompatible polymer approved by the FDA, which recommends it for biomedical applications, such as tissue engineering, tissue regeneration and drug delivery [28]. However, the hydrophobic nature of PLA and the fast degradation of PVA restrict their applications for biological systems. Thus, there is still a need to refine PVA- or PLA-based NFs for tissue engineering and wound dressing, and thus to obtain NFs mats of desirable mechanical, biodegradability and biocompatibility properties. Some studies used thermal treatment to functionalize the surface of PLA meshes with hydrophilic polymers, such as PVA, after immersing them in PVA solution [29]. Obviously, the mechanical properties of PLA coated with PVA have been improved by diminishing its drawbacks of rigidity and poor tensile strength, where it gained more ductility and showed an improvement in its tensile strength. Cell proliferation and attachment have been also enhanced by coating PLA NFs with PVA [29]. Besides, core-shell fibers constitute from PLA as an outer thin coating shell and PVA as an inner hydrophilic swellable core, have been co-electrospun as a sustained and continuous release platform [29]. Incorporation of nCeO_2_ in an appropriate wound dressing membranes or NFs is still under developing. For instance, electrospun poly(3-hydroxybutyrate-co-3-hydroxyvalerate) membranes with more than 1% nCeO_2_ was proved as a strong potential diabetic wound dressing through promoting cell adhesion, proliferation and blood vessels formation [30].

Previous studies cared to develop and design appropriate drug-delivery systems displaying topical and sustained drug-release patterns for diabetic wound healing. Sustained-release dosage is highly recommended during the healing of diabetic wounds, as it maintains the optimal drug concentration at the target site, prevents an undesirable high concentration and leads to the steady and continuous release of drugs during the long-term of treatment [1,31,32,33].

There is still a crucial need to find an appropriate carrier for the topical delivery of nanoceria, particularly in diabetic wound healing. Some recent studies developed nCeO_2_ loaded into monolayer-based membranes that were composed of polymers or polymer blends for chronic diabetic wounds [34,35]. However, such monolayer based patches could not ensure the sustained release of nCeO_2_ during the healing of diabetic wounds which continue for weeks, months or years.

The present study involves, for the first time, to our knowledge, the design and development of electrospun tri-phasic nCeO_2_-loaded PLA/PVA/PLA layer-by-layer NFs, where the PVA is electrospun as a middle NF layer coated by bilayer from PLA. As the study aims to create a ROS free environment on the wound site, thus helping maintain skin-cell regeneration, nCeO_2_-loaded trilayer NFs would be deemed as promising wound-dressing scaffolds. The proposed trilayer NF mats would have the capability to control wound exudate through PVA [36] and to prevent its unpleasant fast degradation via coating it with outer bilayer from PLA. Moreover, sandwiching PVA in outer layers from PLA will ensure good flexibility and biocompatibility and hinder the initial burst release of the loaded bioactive cargo, as well as hydrophobicity, which minimizes its adherence to the wound of the NFs mats [37,38]. The relatively low degradation rate of the outer PLA NFs layers will avoid the burst release of nCeO_2_ from the inner PVA layer in biological environment and will allow its prolonged release, as this ROS scavenger (nCeO_2_) is highly required to be released over weeks, particularly in chronic diabetic wounds. Keeping the desirable concentration of nCeO_2_ in the wound area will help skin cells to grow and proliferate normally, without any destructive effect from the ROS. The chosen set of experiments is intended to pave the way for potential future applications of the developed trilayer-based NFs as nCeO_2_ releasing wound dressing for the healing of diabetic wound.

## 2. Materials and Methods

### 2.1. Materials

Cerium nitrate hexahydrate (Ce(NO_3_)_3_·6H_2_O) that was obtained from Sigma-Aldrich (Darmstadt, Germany), with a purity exceeding 99%, was used as the precursor material. Polyvinyl alcohol (PVA) with molecular weight (Mw = 85,000–124,000) and 87–89% hydrolyzed was purchased from Sigma-Aldrich (Darmstadt, Germany). PLA (molecular weight (Mw) = 420,000 g/mol and polydispersity = 1.5) was received from NatureWorks LLC (Minnetonka, MN, USA). Dichloromethane (DCM) and dimethylformamide (DMF) were purchased from Fisher Scientific, Atlanta, GA, USA. All other chemicals were of analytical grade and were used without further purification. MilliQ water (18.2  Ω cm^−1^) was used to prepare all aqueous solutions.

### 2.2. Synthesis of the Cerium Oxide Nanoparticles (nCeO_2_)

Cerium nitrate (Ce(NO_3_)_3_·6H_2_O) was used as the precursor of nCeO_2_. Nanoceria was synthesized as previously reported [39]. Briefly, cerium nitrate solution was prepared and stirred vigorously for 30 min. Aqueous ammonia was added slowly to the cerium nitrate solution, until light yellow precipitate was formed. This was stirred by using a magnetic stirrer. The nanosuspension was centrifuged at 8000 rpm to separate the nanoparticles (NPs). The centrifugate was washed several times, alternatively with deionized water, and the pellet was dried in an oven, at 60 °C, overnight, and re-dispersed in water for further use.

### 2.3. Physicochemical Characterization of Cerium Oxide Nanoparticles

The absorbance spectra of nCeO_2_ were recorded by UV–Vis spectrophotometer (Shimadzu UV-3600, Kyoto, Japan). Fourier transform infrared (FTIR, JASCO 4700, Portland, OR, USA) analysis was performed in the range of 4000−400 cm^−1^. The purity and crystallinity of the samples were characterized by X-ray diffraction (XRD) analysis, using a MiniFlex X-ray diffractometer in the 2θ range of 20–100°, where the background planes were removed. The crystallite size of samples, was estimated from XRD patterns by applying full-width half-maximum (FWHM) of peaks crystal planes, using the Debye–Scherrer equation (Equation (1)).

*D* = *Kλ*/*β cos θ* (1)

where *D* is the crystallite size, *K* is the Scherrer’s constant (*K* = 0.94), *β* corresponds to the FWHM of the selected XRD peak expressed in radians and *θ* is the selected diffraction peak position.

Scanning electron microscope coupled with energy-dispersive X-ray spectroscopy (SEM–EDX; SEM, MA-EVO10, ZEISS, Oberkochen, Germany) was employed to display the morphology and distribution of the as-prepared nCeO_2_ in powder form and aqueous suspension (1 mg/mL) each. Size and morphology of nCeO_2_ were investigated with TEM for imaging acquisition at accelerating voltage of 100 kV. TEM samples were prepared by depositing 10 μL of the colloidal suspensions on a 200 mesh formvar/carbon film-coated Cu grid, followed by air-drying. Excess film was removed with absorbent paper.

### 2.4. Preparation of the Electrospinning Solutions

PLA/PVA/PLA and PLA/PVA-nCeO_2_/PLA NFs membranes were fabricated by using the electrospinning technique. A 12% (*w*/*v*) PVA solution was prepared in distilled water (DW) with heating at 90 °C and stirring for 4 h. The polymer solution was then left for cooling. After that, different amounts (0.5, 1 and 2%) of nCeO_2_ were added to the polymer solution and left under stirring for 2 h to obtain a homogenous PVA/nCeO_2_ solution. At nCeO_2_ of 1 and 2%, the PVA concentrations were optimized at 11 and 10%, respectively, in order to be well-electrospun. PLA solution (8% *w*/*v*) was prepared in DCM/DMF (7:3) and then left for stirring overnight. Sample formulations were designated as stated in Table 1.

### 2.5. Rheological Properties

The conductivity and viscosity of PVA/nCeO_2_ blends at the different ratios were recorded at room temperature, using an electroconductivity meter and a digital viscometer (DV-E, Brook- field AMETEK, Middleborough, MA, USA), respectively. Each measurement was repeated three times, and the average value was calculated.

### 2.6. Fabrication of nCeO_2_-Loaded PLA/PVA/PLA Electrospun Trilayer Membranes

**Scheme 1:**
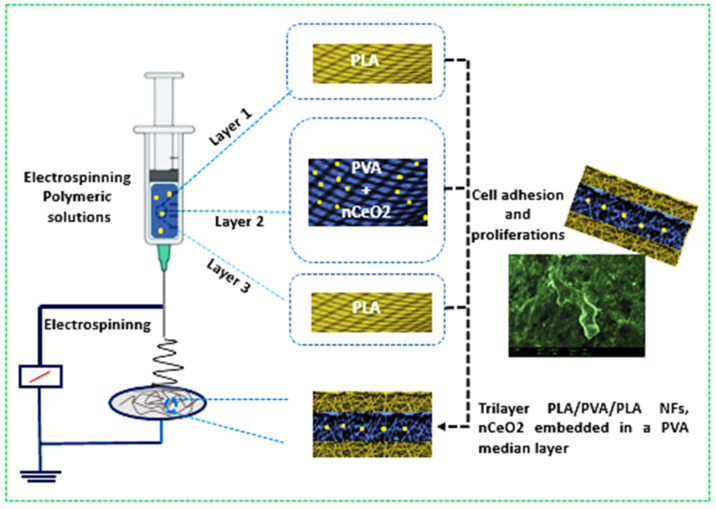
Schematic representation of the preparation and optimization stages of the fabrication of the developed trilayer composed of PLA/PVA/PLA, whereas nCeO_2_ was embedded in a PVA middle layer.

The trilayers composed of PLA/PVA/PLA incorporating nCeO_2_ were fabricated as shown in Scheme 1. The prepared PLA (10 mL) was firstly electrospun at a tip to collector distance of 12 cm. The applied flow rate and voltage were 0.2 mL/h and 21 kV, respectively. The electrospinning of PLA was left for a determined time to form the first outer layer. The thick second middle layer was fabricated by electrospinning of PVA/nCeO_2_ hybrid solution with the same parameters as in the first outer layer but for longer period (duplicate). Finally, PLA solution was electrospun to possess the third outer thin layer with the same period and parameters as in the first outer layer. Fibrous membranes deposited on the collector were carefully removed and stored in a desiccator, at room temperature, until further use. Bare (nCeO_2_ free) electrospun PLA/PVA/PLA NFs membranes were named pure-F1. Membranes with 0.5, 1 and 2% (*w*/*v*) loaded nCeO_2_ were assigned the names nCeO_2_-F2, nCeO_2_-F3 and nCeO_2_-F4, respectively.

### 2.7. Mechanical, Chemical, Surface and Morphological Characterizations

The chemical composition of the prepared samples was studied by FTIR spectroscopy (JASCO 4700, Jasco Manufacturing, Portland, OR, USA). The spectra were recorded over a wavenumber range of 4000–400 cm^−1^ at a resolution of 4 cm^−1^. Differential scanning calorimetry (DSC) was used for each nCeO_2_-loaded NFs, along with the plain NFs to display their thermal behavior and properties, such as glass transition temperature (Tg) and melting temperature (Tm). It was monitored by using (DSC; a Shimadzu DSC-60 Plus machine). Then 10–12 mg sample was sealed in aluminum pans and heated in the range between room temperature and 250 °C at a heating rate of 10 °C per minute, in the presence of nitrogen as an inert carrier gas flowing at the rate of 25 mL/min.

To demonstrate the tensile strength, samples were cut into dimensions of 3×1 cm, then the thickness of the electrospun matrices was measured by using a digital micrometer (C/N 293/100, Mitutoyo, Japan) with ±0.0001 mm accuracy. Mechanical tests were performed by recording the maximum force, elongation at break and elastic modulus during extension at 5 mm min^−1^ by Instron 4411 mechanical tester (Norwood, MA, USA). Tensile strength was calculated by dividing the applied maximum force by the initial cross-sectional area of the sample. Three measurements were performed for each sample.

The morphology of the prepared electrospun NFs was observed under a scanning electron microscope (SEM, MA-EVO10, ZEISS, Oberkochen, Germany), at an accelerating voltage of 20 kV. Samples were cut and coated with gold (120 s) before imaging. Fibrous diameters were visualized by ImageJ, using SEM images. SEM images were analyzed with image analysis software (ImageJ, National Institutes of Health, Bethesda, MD, USA) to determine the average fiber diameters.

### 2.8. Water Absorption and Biodegradability

The water retention and swelling behavior of the prepared nanofibrous mats were weighed (*Wd*) and placed in a phosphate buffer solution (PBS, pH 7.4), at the physiological temperature, 37 °C, through predetermined times up to 72 h. The samples were left in a shaking incubator at room temperature. At specific time intervals, the samples were removed outside the buffer, plotted with a dry filter paper to remove any excess PBS and weighed again (*Ws*), until no change in weight was observed. The swelling percentage was calculated according to Equation (2) [40].

(2) $$\mathrm{Swelling}\;\%\;\left(\%\mathrm{water}\;\mathrm{uptake}\right)=\left[\frac{Ws-Wd}{Wd}\right]\times 100$$

Biodegradability was studied by placing samples of NFs in pH 7.4 PBS, in a shaking incubator, for 21 days. The media were changed and replaced by fresh media every day. The sample discs were withdrawn from the PBS washed with distilled water and dried at 50 °C in an oven, till constant weight was obtained. The weight remaining percentage was monitored daily, using Equation (3), where *Wi* is the initial weight and *Wf* is the final weight measured each day [40].

(3) $$\mathrm{Remaining}\;\mathrm{weight}\;\%\;=\left[\frac{Wf}{Wi}\right]\times 100$$

### 2.9. Encapsulation Efficiency and In Vitro Drug Release

A standard calibration curve was plotted within the range of 0.2, 0.4, 0.6, 0.8 and 1 mg/mL of nCeO_2_ in PBS. The calibration equation was estimated. Nanoceria content in the nanofiber was quantified via spectrophotometer through the complete dissolution of a certain weight of fibers in a solvent and then filtrated. Encapsulation efficiency (EE%) was calculated by using Equation (4).

(4) $$\mathrm{Entrapment}\;\mathrm{efficiency}\;\left(\%\right)=\left[\frac{\mathrm{Measured}\;\mathrm{drug}\;\mathrm{content}}{\;\mathrm{Theoretical}\;\mathrm{drug}\;\mathrm{content}}\right]\times 100$$

The release profile of nCeO_2_ from the trilayer electrospun membranes was studied in phosphate buffer saline (PBS, pH = 7.4) solution, as previously reported [39,41]. The nCeO_2_-loaded NFs (20 mg) were placed in 5 mL of PBS solution, at 37 °C, with constant shaking. At determined time intervals over 7 days (168 h), 1 mL of sample was taken from the release medium and replaced with fresh PBS to maintain the original volume. The amount of nCeO_2_ released at different time intervals in PBS solution was measured by a UV spectroscopy (Shimadzu UV-3600, Kyoto, Japan) [39]. Then, the released concentrations of nCeO_2_ at the different time intervals were monitored and calculated by using Equation (5). Each nCeO_2_-loaded NFs mat was tested in triplicates, and the cumulative release percentage was plotted against time.

(5) $$Cn=Cn\;means+A/V\overset{n-1}{\underset{S=1}{\sum }}Cs\;means$$

where *Cn* is the expected nth sample concentration, *Cn means* is the measured concentration, *A* is the volume of withdrawn aliquot, *V* is the volume of the dissolution medium, *n* − 1 is the total volume of all the previously withdrawn samples before currently measured sample and *Cs* is the total concentration of all previously measured samples before the currently measured sample.

### 2.10. Cell Culture Assays

#### 2.10.1. MTT Cytotoxicity Assay

The cytotoxicity of the produced nanofibers mats was evaluated by using indirect contact MTT assay based on the procedure adapted from the ISO10993-5 standard test method [42,43]. The mouse-embryo-fibroblast 3T3 cell line was obtained from American Type Culture Collection (ATCC). Cells were cultured in Dulbecco’s modified Eagle medium (DMEM, Gibco, Amarillo, TX, USA) with 10% fetal bovine serum (FBS, Gibco, Amarillo, TX, USA) and 1% penicillin/streptomycin, in a 5% CO_2_ humidified air incubator, maintained at 37 °C. When the cells reached 80% of confluence they were washed with PBS and trypsinized with 0.25% Trypsin-EDTA for passaging and seeding each time. The confluent cells were used in cytotoxicity tests and SEM investigations. First, the conditioned medium was prepared to understand any possible toxic effect induced by possible ionic leach-out product from the samples into the medium. For this aim, 5 mL fresh medium was added in tubes with a piece (~0.05 g) of tested material (plain and nCeO_2_-loaded trilayer electrospun membranes), and the tubes were kept in the incubator. After 1 day and 3 days, the conditioned medium was extracted, and then it was later used in cytotoxicity tests. MTT assays were performed in 96-well plates. Then the 3T3 cells (about 10^5^ cells per well) were seeded onto the 4-h UV-sterilized polymers and incubated for 72 h. Cell viability was measured by determining mitochondrial NADH/NADHP–dependent dehydrogenase activity, which resulted in the cellular conversion of the 3-(4, 5-dimethylthiazol-2-gl)-5-(3-carboxymethoxylphenyl)-2-(4-sulfophenyl-2H) tetrazolium salt into a soluble formazan dye. After 1 day and 3 days, supernatants were removed, and 10 µL 3-{4, 5-dimethylthiazol-2yl}-2,5-diphenyl-2H-tetrazolium-bromide (MTT, 5 mg/mL, Sigma) solution was added to each well. Following incubation at 37 °C for 3.5 h and kept in the dark, in a humidified atmosphere, at 5% CO_2_, in air. MTT was taken up by active cells and reduced in the mitochondria to an insoluble purple formazan granule (Mosmann, T., 1983, Rapid colorimetric assay for cellular growth and survival: Application to proliferation and cytotoxicity assays, J. Immunol. Meth., 65, 55–63). Subsequently, supernatant was discarded, and the precipitated formazan was dissolved in dimethyl sulfoxide (100 µL per well), and optical density of the solution was evaluated by using a microplate spectrophotometer (Kayto RT-2100C) at a wavelength of 570 nm.

#### 2.10.2. SEM Investigation

The samples were placed in the wells of 6-well cell-culture plates and sterilized for 4 h by UV. Moreover, 3T3 cells were seeded in these plastic dishes and incubated for 3 days in a humidified incubator at 37 °C with 95% air and 5% CO_2_. At the end of 3 days, the media were removed, and specimens were fixed with 3% volume fraction of glutaraldehyde, subjected to graded (30–100%) alcohol dehydration and kept at −20 °C. Dried mats were sputter-coated with Au and examined by using SEM with 10 kV.

#### 2.10.3. Fluorescence Imaging

The samples were placed in the wells of 6-well-cell culture plates and sterilized for 4 h by using UV. Then, the 3T3 cells were seeded in these plastic dishes and incubated for 3 days in a humidified incubator, at 37 °C, with 95% air and 5% CO_2_. At the end of 3 days, the medium of the cells was removed, and specimens were fixed with 3% volume fraction of glutaraldehyde. We prepared the dye to be 1 μg/mL (dissolve with PBS), placed 100 μL of DAPI in each sample and waited 10 min. After 10 min, we washed with 1X PBS. The samples were subjected to graded (30–100%) alcohol dehydration. At the end of this step, samples were taken on the slide. We put 2 or 3 drops of Fluoromount and closed the coverslip for 30–45 min, until dry. At the end of the time, the samples were packaged in a way that did not receive light and were then placed in +4 °C. Finally, the DAPI solution was removed, and the constructs were taken for imaging under fluorescence inverted microscope (Leica). Standard deviations were used to determine the error bars.

### 2.11. Statistical Analysis

The results were analyzed by using GraphPad Prism 5 software (version 5.01). The statistical comparison was carried out by using Student’s *t*-test, with a *p*-value used to indicate significance.

## 3. Results and Discussion

### 3.1. Characterization of nCeO_2_

**Figure 1:**
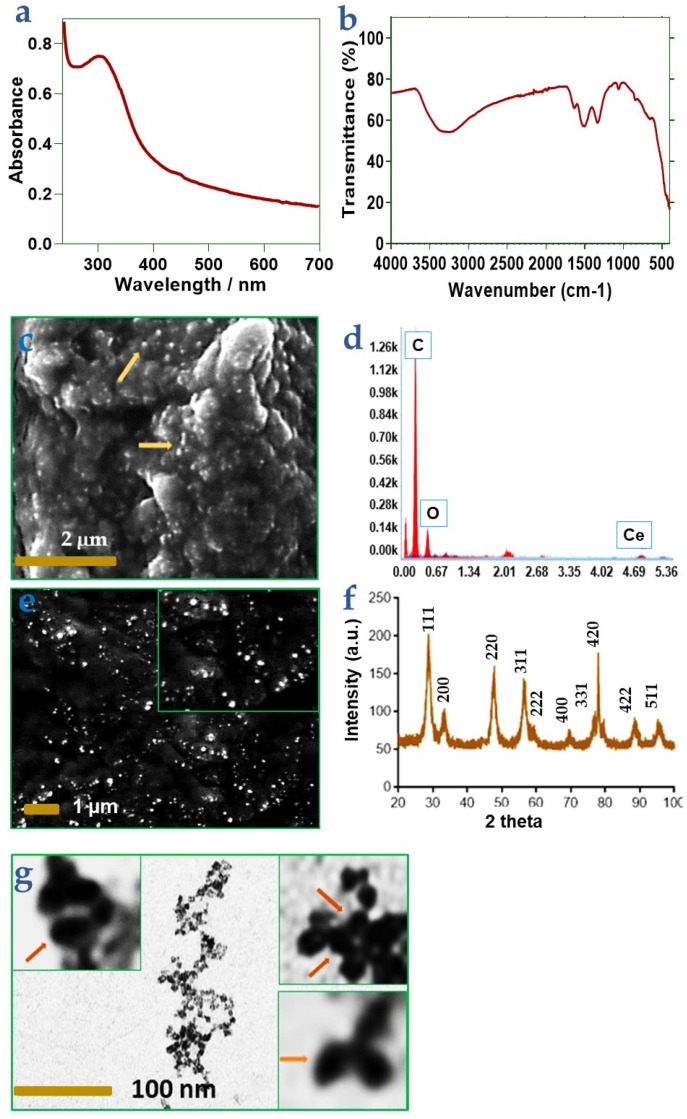
Characterization of nCeO_2_, using (**a**) UV spectra, (**b**) FTIR, (**c**) SEM–powder NPs, (**d**) EDX, (**e**) SEM with NPs suspended in aqueous solution, (**f**) XRD and (**g**) TEM micrograph, where the inserts declare the spherical/ovoid-shaped nCeO_2_.

The absorbance spectra of dispersed nCeO_2_ in water were recorded and are shown in Figure 1a. It was observed that absorbance peak locates at around 303 nm. This result is in accordance with other reports [44,45,46] recorded the UV spectra of nCeO_2_ in the region of 300–340 nm.

Figure 1b shows the IR spectra of nCeO_2_ powder in the range of 4000–400 cm^−1^. The broad absorption peak that appeared at 3253 cm^−1^ has a corresponding –OH vibration. The spectra exhibited absorption bands at 441 and 622 cm^−1^, and they could be used as a detection for the stretching band of Ce-O. The bands at 1060 and 1332 cm^−1^ are associated with H_2_O bending vibration. Due to H-O-H flexion, it produces a band at 1629 cm^−1^ that overlaps with the band attributed to the O-C-O stretching [45,47,48,49].

The morphology of nCeO_2_ in powder form was demonstrated by using SEM and TEM, as shown in Figure 1. The SEM micrograph (Figure 1c) confirmed the synthesis of uniform nCeO_2_ NPs. The dispersed nCeO_2_ NPs in aqueous solution (1 mg/mL) as shown in Figure 1e was also depicted by SEM, and it shows that the NPs produced without aggregation were well distributed. EDX provides information on the composition of the NPs. Figure 1d displays the presence of Ce and O as depicted in the EDX spectrum and it indicated the presence of pure cerium oxide NPs.

The size and shape of the as-synthesized nCeO_2_ were investigated with the aid of TEM. The transmission electron micrographs (Figure 1g) depicted that the formed nCeO_2_ NPs are spherical/ovoid-shaped NPs with an average size of 17.0 nm.

The XRD pattern of nCeO_2_ is shown in Figure 1f. The XRD pattern was scanned from 20–80 degrees with the scan rate 2θ min^−1^. The XRD profile confirmed the polycrystalline nature of the nCeO_2_. The high intensity peaks were observed at 28.15, 33.07, 47.19, 56.29, 59.36, 69.73, 77.01 78.84, 88.73 and 96.61, and they correspond to the (111), (200), (220), (311), (222), (400), (331), (420), (422) and (511) crystal planes, a cubic structure fluorite of CeO_2_. The crystal planes were in good accordance with JCPDS No. 34-0394 of CeO_2_ crystal. The diffraction peaks in these XRD spectra indicate the pure cubic fluorite structure. Such findings are similar to those in a previous study [49]. The average crystallite size was calculated from the full-width at half maximum (FWHM) of the diffraction peaks corresponding to the (111), (200), (220) and (311) diffraction planes by using the Debye–Scherrer formula. Scherrer analysis showed an average crystallite size at 16.3 ± 1.4 nm, which is consistent with the TEM micrograph.

### 3.2. Viscosity and Conductivity

The measured values of viscosities were used to record the absolute viscosities over different concentrations: 12/0.5, 11/1 and 10/2 of PVA/nCeO_2_ blends (Table 2). Moreover, it was observed that the viscosity of nCeO_2_/PVA blends was increased by increasing the proportion of the nanoceria, and this was likely attributed to the potential crosslinking through the interaction of hydroxyl groups of PVA and nanoceria; thus, the viscosity was increased. As can be noted from Table 2, the conductivity was increased by increasing the amount of nCeO_2_. This could be attributed to the calcinated nCeO_2_ [50].

### 3.3. SEM of Pure and nCeO_2_-Loaded Trilayer NFs

**Figure 2:**
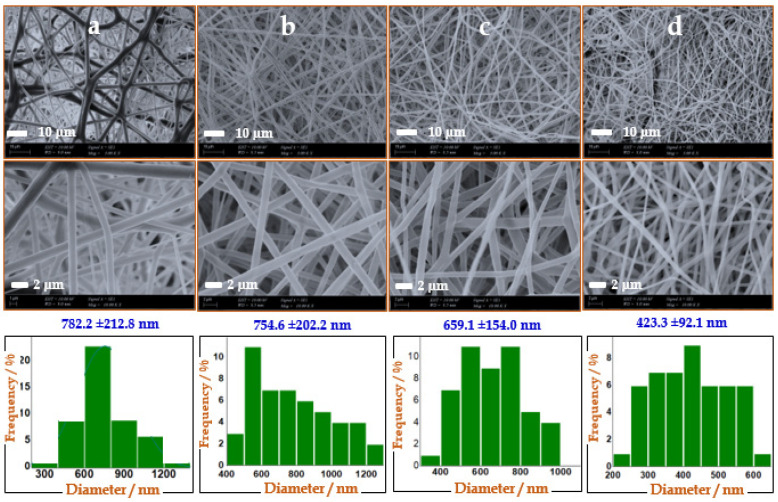
SEM images and diameters histograms of the electrospun nanofibrous trilayer membranes: (**a**) pure-F1, (**b**) nCeO_2_-F2, (**c**) nCeO_2_-F3 and (**d**) nCeO_2_-F4.

The morphology of the produced NFs was observed using SEM, as shown in Figure 2. It was found that the fibers diameters were decreased by increasing the embedded Ce NPs. Plain NFs showed an average diameter of 792 nm, whereas averages diameters of trilayers/nCeO_2_-F1, trilayers/nCeO_2_-F2 and trilayers/nCeO_2_-F3 were recorded at 782.2, 663.3 and 423.3 nm, respectively. Moreover, NFs appeared more uniformly by increasing the loading of nCeO_2_ NPs. Such results were consistent with a previous study which demonstrated that the average diameter of NFs were reduced by increasing the loading amount of nCeO_2_ up to 5% [51]. Moreover, the higher content of NPs leads to higher in the conductivity (more charges on the jets) and viscosity, with deposition of thinner NFs. The solution with higher viscosity is ejected hard from jets [52].

### 3.4. Tensile Strength

**Figure 3:**
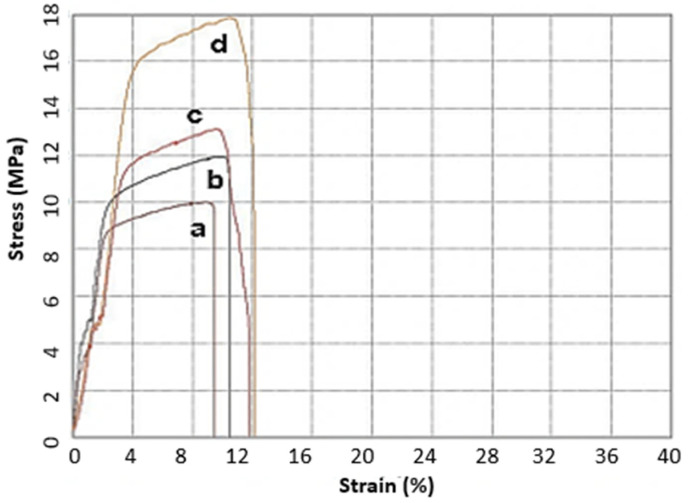
Mechanical properties of (a) pure-F1, (b) nCeO_2_-F2, (c) nCeO_2_-F3 and (d) nCeO_2_-F4.

The tensile strength was carried out to characterize the mechanical properties of the electrospun NFs. The tensile property is important to evaluate the applicability of the wound dressing where a good wound dressing mats could be characterized by a sufficient flexibility [53]. As observed in Figure 3, the developed NFs exhibited the tensile properties of the plain and nCeO_2_-loaded NFs through elongation at break. Plain trilayer membranes displayed tensile strength around 9.9 MPa, with elongation at break of 9.4%. It could be observed that, by increasing the content of nCeO_2_, both tensile strength and elongation at break were increased. The three nCeO_2_-loaded electrospun NF membranes, namely trilayers/nCeO_2_-F2, trilayers/nCeO_2_-F3 and trilayers/nCeO_2_-F4, showed tensile strengths around 12, 12.7 and 17.8, respectively. Additionally, their elongations at breaks were around 10.4, 11.1 and 12.2%, respectively. Thus, loading 1 and 2% of nCeO_2_ in the produced electrospun NF membranes enhanced their mechanical properties and thus allowing them to be used as wound-dressing nanofibrous mats.

### 3.5. Swelling and Biodegradability

**Figure 4:**
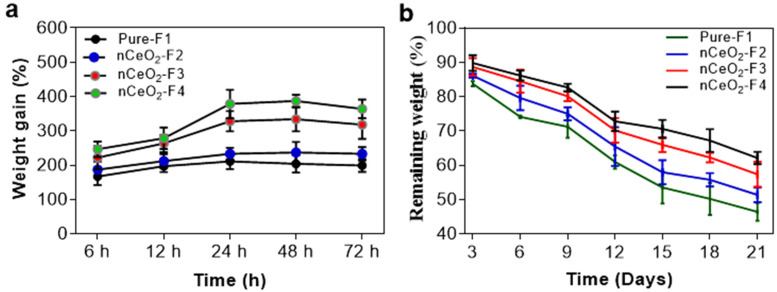
(**a**) Swelling and (**b**) biodegradability of the developed nCeO_2_-loaded NFs.

The swelling and absorbing capacity of the prepared NFs mats will give a close-up of its ability to absorb exudates and provocative fluids. A wound dressing that can offer a good swelling capacity to absorb excessive exudates and fluids secreted by the wound will be able to accelerate the healing process. The water absorption of nCeO_2_-F4 was the highest among all the tested nanofibrous mats. This can be attributed to the higher content of the hydrophilic cerium NPs. Upon immersing nCeO_2_/NFs in PBS solution, it swells along with creating tiny pores within the body of fibrous mats, and this is likely to be a good physical property for wound healing and may ensure their potential application in wound dressing [54]. Capability of NFs to retain water can keep the dressing wet and thus protect the skin from dehydration [55]. As noted in Figure 4a, 1 and 2% nCeO_2_-loaded NFs could significantly enhance the swelling ratio around 318 and 364% of the initial dry weight, respectively. The swelling ratios of nCeO_2_-loaded NFs, namely nCeO_2_-F2, nCeO_2_-F3 and nCeO_2_-F4, were found to be approximately 1.2-, 1.6- and 1.9-fold of that of the native NFs, respectively. Presence of PVA as a middle layer could improve the swelling property of the developed nanofibrous membrane, where PVA has a good capacity for water uptake [56].

An in vitro biodegradability assessment of both pure and Ce NPs-loaded NFs (Figure 4b) was performed for 21 days in PBS (pH 7.4) to simulate the actual environment of skin wound healing. The weight remaining % of samples was monitored, calculated and plotted along 21 days, at certain time points, as documented above. The study showed that both loaded and non-loaded NFs mats could withstand for a considerable period of time to perform their function as a wound dressing before being degraded. It was also detected that the pure NFs lost around 54% of their weight during the 21-days study. Moreover, the weight remaining % increased by increasing the loading of NPs, as it was observed that nanofibrous mats loaded with 0.5, 1 and 2% Ce NPs exhibited around 51, 57 and 62% remaining weights, respectively. The -OH groups in PVA could have hydrogen interaction with the surface of metal NPs. This hydrogen bonding could stabilize the nanofibrous mats and delayed their degradation rate [57].

### 3.6. FTIR Study

**Figure 5:**
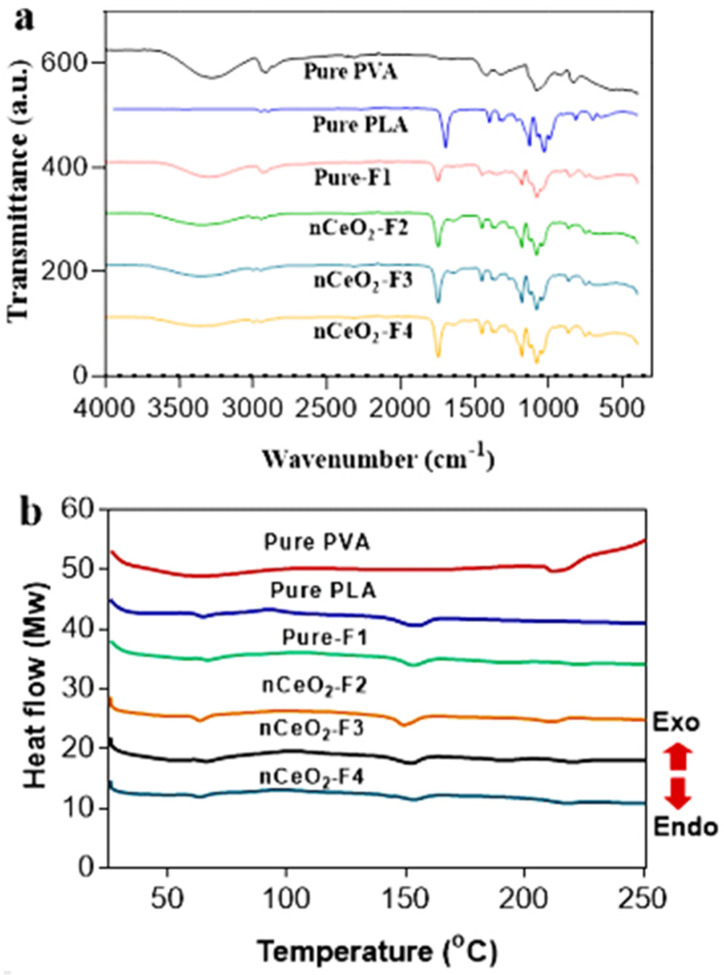
FTIR spectra (**a**) and DSC thermograms (**b**) of the nanofibrous membranes under study.

The chemical compositions of the nCeO_2_-loaded PLA/PVA/PLA NFs were confirmed through an FTIR investigation (Figure 5a). The main characteristic peak of the plain PVA NFs is shown at 3200–3680 cm^−1^ (precisely at 3289 cm^−1^) which is attributed to the stretching vibration of –OH group. The peak at 2923 cm^−1^ is corresponding to C–H stretching. The absorbance peak at 1417 cm^−1^ is assigned to bending of –CH_2_, whereas the band at 1077 cm^−1^ is corresponding to stretching of C–O.

For the PLA NFs, the characteristic needle-like peak observed at 1747 cm^−1^ is corresponding to the carbonyl stretching (–C=O), while the band at 1081 cm^−1^ is associated with the stretching of C–O [58].

It can be observed that pure-F1 showed most of the peaks of both PVA and PLA with slight changes in intensities and band-shifts, confirming that all characteristic peaks of the polymeric constituents (PVA and PLA) were recorded. On the other hand, the band-shifts, broadening, with low intensities of –OH peak of PVA at 3200–3680 cm^−1^ in nanoceria-loaded NFs could be attributed to the hydrogen bonding between NPs surface and –OH group of PVA. Furthermore, the Ce-O interaction band at 400–600 cm^−1^ was noted in nanoceria-loaded NFs.

### 3.7. Differential Scanning Calorimetry Studies

In Figure 5b, it can be observed that the glass transition (Tg) and melting temperature (Tm) for pure PVA were recorded at 64 and 214 °C, respectively. Meanwhile, in PLA, both Tg and Tm were detected at 65 and 154 °C, respectively. The pristine electrospun trilayer nanofibrous membrane depicted Tg at 66 °C, whereas its Tm was observed at 153 °C. These peaks were not similar to the coaxial core–shell electrospun ones of PVA/PLA in a previously reported study [59], due to the difference in the applied electrospinning technique and the deposition of the NFs. The Tm of the loaded NFs in nCeO_2_-F2, nCeO_2_-F3 and nCeO_2_-F4 was observed at 151, 153 and 154, respectively. There is a second melting temperature attributed to the presence of PVA, and it is red-shifted and less prominent in pure-F1, nCeO_2_-F3 and nCeO_2_-F4, due to sandwiching of a middle PVA layer into the PLA bilayer, in addition to the increase in thermal stability by incorporation of nanoceria at 1 and 2%. The peak of the second melting temperature is a bit more prominent in case of nCeO_2_-F2, which indicates their low thermal stability than nCeO_2_-F3 and nCeO_2_-F4. This peak can be observed at 213, 219 and 219 in nCeO_2_-F2, nCeO_2_-F3 and nCeO_2_-F4, respectively. Such findings confirm the electrospinning of the trilayers composed of nanoceria-loaded PVA as a middle layer coated by PLA as outer bilayer. The obtained results, in general, demonstrate that both nCeO_2_-F3 and nCeO_2_-F4 showed more thermal stability than nCeO_2_-F2, where the thermal stability is better by incorporation of nanoceria up to 1 and 2%, as opposed than 0.5%. By incorporating high amount of nCeO_2,_ the three layers are getting more fused and stable than using a low amount. This may be explained by the fact that well-dispersed inorganic Ce NPs loaded in polymeric NFs in the case of nCeO_2_-F3 and nCeO_2_-F4 could retard the diffusion of oxygen to NFs mats and acting as a heat barrier accompanied with retarding the escape of volatile products and eventually leading to higher thermal stability of NPs-loaded NFs [60]. As a result of strong interfacial interactions through hydrogen bonding between metal NPs and the –OH group of PVA, it produced a stabilized product with high thermal stability [61].

### 3.8. Drug-Release Study

The trilayer-based NFs were formed in order to probe drug-release patterns of nanoceria from the PLA/PVA-nanoceria/PLA. For such assay, nanofibrous mats were incubated at 37 °C in a release buffer (pH 7.4), and samples were collected and replaced with fresh buffer at the predetermined times for about 168 h (7 days). The absorbance of the collected samples was measured at 317 nm and compared to a standard curve of nCeO_2_ in order to determine the percent of the Ce NPs released over time. It seemed that water molecules diffuse across the PLA outer layers to the middle layer of the PVA/Ce NPs hybrid mat. The solubilized PVA/nCeO_2_ then diffused through the PLA layers to the buffer media. At higher nCeO_2_ contents, the complexed PVA/nCeO_2_ was slowly solubilized, and it was sustained-released from PLA over a longer period.

The mechanism of drug release from electrospun NFs is influenced by the polymer composition. There are four kinds of drug-release mechanisms: drug diffusion, drug dissolution, drug adsorption–desorption and polymer erosion/degradation. For non-degradable polymers, the drug release follows diffusion-controlled kinetics; meanwhile, it follows degradation-controlled kinetics in the case of fast degradable polymers. The combined kinetics of diffusion and degradation are found in slowly degradable polymers [62,63].

PLA is characterized by hydrolytic degradation through four main stages, starting with the diffusion of water molecules, followed by hydrolysis with formation of oligomers. By the third stage, oligomers diffuse out of the polymer matrix and leave many porous regions in the body of the polymer matrix. Thus, the loaded drug is sharply released through that porous structures, and the release continues with the proceeding of a homogenous slow degradation rate of the porous polymer that composes the fourth stage.

The trilayer NF mats in the current study were designed in such a way as to reduce the burst release and confer a sustained release of CeO_2_, whereas the hydrophobic bilayer of PLA were optimized to coat the nCeO_2_-loaded PVA middle layer. In the present study, it started with the degradation kinetic from the core middle PVA layer, which was then followed by diffusion kinetics through the outer non-degradable layers of PLA. At a higher nCeO_2_ content, the complexed PVA-nCeO_2_ was relatively slowly degradable; thus, the nCeO_2_ release followed the combined diffusion/degradation kinetics in the middle layer, and then the diffusion kinetics through the outer PLA bilayer followed.

**Figure 6:**
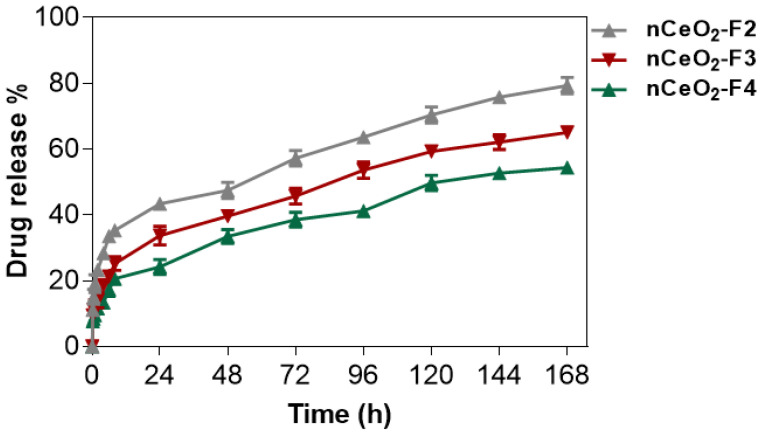
Percentages of cumulative drug release of nCeO_2_ from nCeO_2_-F2, nCeO_2_-F3 and nCeO_2_-F4.

As observed in Figure 6, nCeO_2_-F4 displayed a minimal burst release of 17% after 6 h, and then followed by a sustained release over 168 h. By the end of this time, approximately 54% of initial amount nCeO_2_ was released. The burst release was moderate in nCeO_2_-F3, as it recorded around 21% with releasing 65% of the initial content of nCeO_2_.

Meanwhile, the electrospun membrane with low loading 0.5% of nCeO_2_ (nCeO_2_-F2) demonstrated the highest burst release (33%). It also displayed a fastest release profile where over 79% of the nCeO_2_ content was released during the period of study. At nCeO_2_-F2, the nanoceria was free and not bound well with polymer; thus, NPs dissociated rapidly with the maximum burst release.

The entrapment efficiency was found to be approximately 91, 96 and 98% in nCeO_2_-F2, nCeO_2_-F2 and nCeO_2_-F2, respectively. These losses might occur mainly during electrospinning. This indicated that conjugation and crosslinking of nCeO_2_ with polymeric solution are proportional to the incorporated amount of nCeO_2_ to form a homogenous solution convenient for electrospinnability with minimum losses for its bioactive materials. The PVA/nCeO_2_ coated with outer bilayer of PLA displayed a controlled release for nanoceria.

Interestingly, the trilayers of a middle PVA coated with bilayer of PLA loaded with 1 and 2% nCeO_2_ were therefore efficient to deliver a low and consistent dose of nCeO_2_ for prolonged times. For such nanofibrous mats, F3 and F4 were characterized by a sustained-release profile and thus can provide the diabetic wound area with the loaded drug (nCeO_2_) in a steady rate over a long period of treatment.

### 3.9. Cyto-Biocompatibility

**Figure 7:**
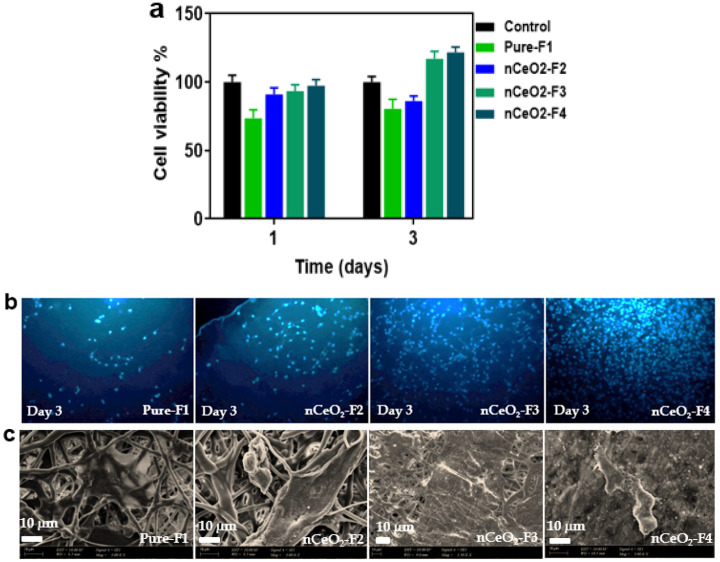
Cell viability using MTT assay (**a**); fluorescence images showing the proliferation of mouse fibroblast cell line (3T3) on the nanofibrous mats, using DPAI staining after 3 days (**b**); and SEM micrographs of cells proliferation and adhesion on the pure and nCeO_2_; loaded nanofibrous mats (**c**).

The biocompatibility of 3T3 mouse embryo fibroblasts (MEF) on the pure-F1, nCeO_2_-F2, nCeO_2_-F3 and nCeO2-F4 after 1 and 3 days was investigated using indirect contact MTT assay (Figure 7a). It was observed that the cell viability on plain-F1 was lower than other loaded NFs, where it displayed 80% cell viability after 1 day of incubation. Meanwhile, the cell viability of nCeO_2_-F2, nCeO_2_-F3 and nCeO_2_-F4 after 1 day was around 91, 93 and 97%. The increase in cell viability for nCeO_2_-F1 after 1 day can be attributed to the burst release of nanoceria, which maintained and enhanced the cell growth and viability. After 3 days, the cell growth and viability on pure-F1 and nCeO_2_-F2 decreased to 73 and 86%, respectively. Meanwhile the cell viability increased to 103 and 122% by seeding the cells over nCeO_2_-F3 and nCeO_2_-F4, respectively. Interestingly, the plain trilayer electrospun membrane (PLA/PVA/PLA) showed no toxic effect on cell viability, and it can be considered as base wound-dressing mats.

As shown in Figure 7b, DAPI staining was also used for visualizing of the increasing and spreading of MEF upon their seeding on plain-F1 and nCeO_2_-loaded membranes for 3 days. The obtained results revealed that the nCeO_2_-F3 and nCeO_2_-F4 showed a slight increase in the proliferated cells compared to nCeO_2_-F2 and non-loaded mats. By increasing of the loaded % of nCeO_2_, the numbers of cells increased. Such cell enhancement and proliferation can be seen in nCeO_2_-F3, but it was more clearly in nCeO_2_-F4.

To evaluate the effect of the nCeO_2_-loaded in the electrospun membranes on cell adhesion, we cultured MEF on the membranes and determined the cell adhesion behavior using SEM. The representative images (Figure 7c) show the cell adhesion characteristics on pure-F1 and nCeO_2_-loaded membranes after 3 days of cell culture. The cells were adhered less on neat-F1 and the membranes loaded with 0.5% nCeO_2_-F2. Interestingly, higher cell adhesion was observed by higher loading nCeO_2_ on the membranes; nCeO_2_-F3 and nCeO_2_-F4.

Higher spreading, adhesion and numbers of cells were observed on nCeO_2_-F3 and nCeO_2_-F4 nanofibrous mats, as compared to the plain membranes. Such findings reveal that the developed nCeO_2_-loaded NFs fabricated in the current study could enhance cells attachments and proliferations; thus, they can be used efficiently for skin regeneration and healing of chronic diabetic wounds.

In previous studies, nCeO_2_ was incorporated in electrospun membranes, but the current study is the first to sandwich nCeO_2_ in PVA as a middle layer coated with two outer layers made of PLA. Such a developed trilayer membrane could act as an efficient drug delivery system to release nCeO_2_ efficiently and in a sustained style over the period of cell seeding. Thus, this continuous release is claimed to maintain nCeO_2_ in a considered concentration in the cell media, and it then could efficiently play its role as ROS scavenger and maintains the cells to grow and proliferate up to 121% after 3 days. This can be cleared in both nCeO_2_-F3 and nCeO_2_-F4 because of their higher loading of nCeO_2_ than nCeO_2_-F2, and both of them showed an EE% and drug-release profile that were higher than in nCeO_2_-F1.

The efficient topical use of nanoceria (nCeO_2_ NPs) for wound healing is enhanced by penetrating nanoceria into wounded tissues and reducing the oxidative damages to the cellular membranes and proteins [16].

## 4. Conclusions

In the present study, nCeO_2_-trilyers-based NFs were successfully synthesized. The as-prepared nanoceria was incorporated into PVA as a middle NF layer, which was coated with outer bilayer from PLA NFs. The conformational physicochemical and morphological properties of the electrospun nCeO_2_-loaded NFs were monitored by FTIR, DSC and SEM. The morphology and the thermal and mechanical properties of PLA/PVA/PLA NFs were improved by the incorporation of a higher amount of nanoceria, rather than a lower amount. The designated trilayer PLA/PVA/PLA NFs could be considered as efficient drug carrier systems for the nanoceria with better cyto-biocompatibility. Trilayer PLA/PVA/PLA NFs loaded with nanoceria (1 and 2%) provided optimum platforms for the cell viability, with a superior support for fibroblasts adhesion and proliferation. Overall, the electrospun trilayer PLA/PVA/PLA-based scaffolds are suitable platforms to integrate CeO_2_, and they can be proposed as a candidate for the treatment of diabetic wounds.

## Figures and Tables

**Table 1:** Composition of the synthesized nCeO_2_-incorporating NFs.

Sample No.	First-Layer Solution (the Bottom Layer)	Second-Layer Solution (the Middle Layer)		Third-Layer Solution (the Upper Layer)	Nanofibers Sample Code
	PLA (*w*/*v*%)	PVA (*w*/*v*%)	nCeO_2_ (*w*/*w*%)	PLA (*w*/*v*%)	
F1	8	12	-	8	Pure-F1
F2	8	12	0.5	8	nCeO_2_-F2
F3	8	11	1	8	nCeO_2_-F3
F4	8	10	2	8	nCeO_2_-F4

**Table 2:** Viscosity and conductivity of PVA/nCeO_2_ blends.

PVA (*w*/*v*%)/nCeO_2_ (*w*/*w*%)	Viscosity (cP)	Conductivity (µS cm^−1^)
12/0	97.1 ± 7.4	834.0 ± 12.7
12/0.5	137.3 ± 8.7	1057.3 ± 17.3
11/1	198.5 ± 9.6	1331.5 ± 21.1
10/2	266.0 ± 11.3	1677.8 ± 24.6
